# Acoustic Sensor Planning for Gunshot Location in National Parks: A Pareto Front Approach

**DOI:** 10.3390/91209493

**Published:** 2009-11-26

**Authors:** Francisco Javier González-Castaño, Javier Vales Alonso, Enrique Costa-Montenegro, Pablo López-Matencio, Francisco Vicente-Carrasco, Francisco J. Parrado-García, Felipe Gil-Castiñeira, Sergio Costas-Rodríguez

**Affiliations:** 1 Fundacion Centro Tecnologico de las Telecomunicaciones de Galicia (Gradiant), Campus As Lagoas/Marcosende, Vigo, Spain; E-Mail: scostas@gradiant.org (S.C.-R.); 2 Telematics Engineering Department, University of Vigo, Spain; E-Mails: kike@det.uvigo.es (E.C.-M.); xil@det.uvigo.es (F.G.-C.); 3 Information and Communications Technologies Department, Technical University of Cartagena, Spain; E-Mails: javier.vales@upct.es (J.V.A.); pablo.lopez@upct.es (P.L.-M.); francisco.vicente@upct.es (F.V.-C.); fco.parrado@upct.es (F.J.P.-G.)

**Keywords:** acoustic sensing, sound location, sensor synchronization, sensor network planning, derivative-free optimization, Pareto front

## Abstract

In this paper, we propose a solution for gunshot location in national parks. In Spain there are agencies such as SEPRONA that fight against poaching with considerable success. The DiANa project, which is endorsed by Cabaneros National Park and the SEPRONA service, proposes a system to automatically detect and locate gunshots. This work presents its technical aspects related to network design and planning. The system consists of a network of acoustic sensors that locate gunshots by hyperbolic multi-lateration estimation. The differences in sound time arrivals allow the computation of a low error estimator of gunshot location. The accuracy of this method depends on tight sensor clock synchronization, which an ad-hoc time synchronization protocol provides. On the other hand, since the areas under surveillance are wide, and electric power is scarce, it is necessary to maximize detection coverage and minimize system cost at the same time. Therefore, sensor network planning has two targets, *i.e.*, coverage and cost. We model planning as an unconstrained problem with two objective functions. We determine a set of candidate solutions of interest by combining a derivative-free descent method we have recently proposed with a Pareto front approach. The results are clearly superior to random seeding in a realistic simulation scenario.

## 1. Introduction

The Spanish SEPRONA [1] agency fights against poaching with considerable success. However, the problem remains relevant. Since 2007, SEPRONA has detained over 150 people for hunting felonies [2, 3]. Consequently, the authors have proposed the DiANa project, *Detección de caza furtiva con Armas de fuego en parques NAcionales* (Detection of illegal hunting with gunfires in national parks), to automatically detect and locate gunshots, which is endorsed by Cabañeros National Park [4].

The DiANa system consists of a network of acoustic sensors that locate gunshots. There exist some commercial solutions for gunshot detection, although they do not locate sound sources [5]. There also exist sound location tools for the military, but they are too costly and cannot be deployed in large numbers in civilian applications [6]. This paper presents an original method for gunshot location and a planning algorithm for its deployment in large terrain extensions (e.g., a national park). Two key design issues are implementation cost minimization and performance maximization in real-time scenarios. Therefore, sensor protocols and tasks (such as time synchronization and gunshot position estimators), as well as network planning for node placement, have been taken into account.

Assuming a group of sensors has correctly detected an acoustic signal (e.g., using Gaussian Mixture Models, GMM [7, 8]), the location of this signal results from the combination of sensor data. A sensor only knows its own position and local time, and therefore these measures must suffice to locate the gunshot.

In the literature there are several location estimators, such as triangulation [9] and trilateration [10] methods. In the triangulation schema, every sensor determines the direction from which the acoustic event is detected, and then the location of that event is calculated as the intersection of the detection directions. We discarded this alternative, because determining the direction of the acoustic event would make the hardware design too complex and costly, and the solution would be highly sensitive to terrain shape. A trilateration schema determines the location of the event from the distance between the source of the acoustic event and a fixed sensor. These distances can be obtained if the generation time of the event is known. However, in our scenario, only the detection time is available to the nodes.

**Figure 1.**
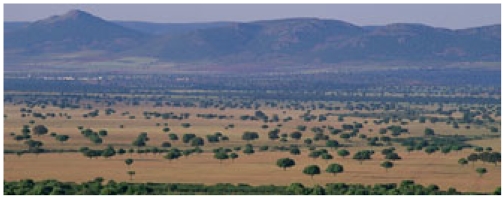
Landscape of the Cabañeros National Park.

A directly observable acoustic signal between a couple of microphones is the time difference of arrival (TDoA) [11–13]. The TDoA technique exploits the relationship between distance and transmission time when the propagation speed is known. Once the time delays are calculated, they are processed in order to estimate the location of the source [14–16]. Due to the distances between the deployed sensors (in the order of hundreds of meters), and the smooth landscape in Cabañeros National Park (Figure 1), we have assumed a two-dimensional scenario.

The hyperbolic location method [17] minimizes an error measure that is a nonlinear function of the potential source location. This approach is scalable, since location accuracy increases with the number of nodes that detect the gunshot (see Section 5.). Its domain can be a plane or a three-dimensional space.

TDoA-based ranging techniques require accurate clock synchronization. Every sensor knows its own position exactly, and it records the arrival time of the sound event. So, the sensor clocks must be as tightly synchronized as possible, using dedicated time-synchronization algorithms. Since a gunshot location network must scale to large sizes, it is necessary to minimize the number of exchanged messages to attain convergence, keeping energy consumption at reasonable levels if the electric grid is not available.

Selecting a synchronization schema for a real application like ours is not easy. The best known synchronization schemas implement network mechanisms to adjust all local clocks to the same value. This is achieved by exchanging time stamps between node pairs. The more frequent the exchanges, the higher the time accuracy. Two representative examples are Reference Broadcast Synchronization (RBS) [18] and the Timing-Sync Protocol for sensor Networks (TSPN) [19]. We discarded GPS receivers due to their high cost. In Section 2.3. we propose a new ad-hoc flood method to set the clock times in every network node to the same value. In this method, the nodes do not exchange synchronization messages, and thus they save power. Once a node detects a gunshot, the time of the event is transmitted to the sink node through a previously generated path. The method performs a cooperative backward time adjustment, so that every node along the path is able to estimate the event time.

Regarding network planning, since the areas under surveillance are wide, and electric power is seldom available, it is necessary to both maximize detection coverage and minimize system cost. Therefore, we model sensor network planning as an unconstrained problem with two objective functions. We provide a set of candidate solutions of interest by combining a derivative-free descent method we have recently proposed and a Pareto front approach.

Due to the inherent difficulties exhibited by the thus far formulated models in sensor network planning, several heuristic optimization strategies have been proposed in the literature: variants of simulated annealing [20], genetic algorithms [21], gradient descent (when applicable) [22] and others [23, 24].

Some of these approaches (simulated annealing, genetic algorithms and the like) do not guarantee theoretical convergence. Regarding gradient descent methods, the gradient is often unavailable or too costly to compute. Therefore, we have adapted a non-monotone derivative-free optimization technique with guaranteed convergence [25] to formulate and solve a computationally efficient **optimization model** with a dual objective: maximization of acoustic network coverage and minimization of power infrastructure cost. The results are presented as a Pareto front, revealing solutions that are clearly superior to random seeding.

Our notation is as follows: Lower case Greek letters are scalars, lower case Latin letters are vectors in *IR*^2^, ${\text{x}}_{k}^{j}$ is the *j-th* component of the vector *x_k_*, and ‖*x*‖ is the Euclidean norm. A capital Latin letter, say *S*, stands for a collection of vectors in *IR*^2^; if *S* = {*s*_1_, …, *s_p_*} we also say that *S* ∈ *IR*^2^*^p^*; *τS* = {*τs*_1_, …, *τ*s*_p_*}, and the sum *Z* = *S* + *D* means that *S* and *D* have the same number of elements, say *p*, and *z_k_* ∈ *Z* if *and only if* ∃*k* ∈ {1, …, *p*} | *z_k_* = *s_k_* + *d_k_*. In general the subindex *i* is the value of an entity (scalar, vector, set, and so forth) at the *i-th* iteration of an algorithm; for instance *S_i_* = {*s_i_*_1_, …, *s_ip_*} is a set of *p* vectors in *IR*^2^, at the *i-th* iteration.

The rest of the paper is organized as follows:

Section 2. describes the sound location and node synchronization procedures. In Section 3. we present the optimization model to plan the acoustic sensor network. In Section 4. we verify that the problem fulfills the conditions in [26] to apply the advanced derivative-free algorithms in [25]. In Section 5. we perform numerical tests to evaluate the approach. Finally, Section 6. concludes the article.

## 2. Gunshot Location

In this section we describe the location procedure for acoustic events, which we have implemented for MicaZ motes. The goal of our system is gunshot location by means of a sensor network. Location is based on hyperbolic positioning [17]. Hyperbolic positioning requires the sensor clocks to be synchronized, in order to apply the TDoA technique. For this reason we have implemented a synchronization protocol in the MICAz motes. Next, we describe the system architecture, the location method and, finally, the synchronization schema.

### 2.1. System architecture

**Figure 2.**
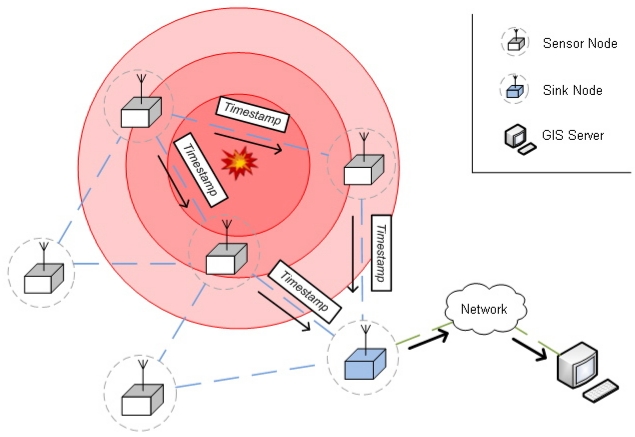
Gunshot location architecture.

Figure 2 shows the system architecture, with three components:

- Sensor nodes: The sensor nodes in known positions are equipped with the necessary hardware for the detection of acoustic events. They can discriminate between *normal* and *shot* segment classes in audio streams. When a sensor node detects a sound event, it transmits a packet with information about the type of sound event and a sound timestamp to a special node, the sink.
- Sink nodes: Sink nodes collect the packets sent by sensor nodes and deliver them to the GIS server to calculate the position of the sound event. Sink nodes may be sensing nodes as well.
- GIS server: Using the information from the sink nodes, the GIS server estimates the position of the acoustic event by means of a hyperbolic method, described in Section 2.2.

### 2.2. Source location procedure

Since our optimization model (see Section 3.) considers a flat landscape with scattered trees, we have selected a two-dimensional hyperbolic positioning algorithm. Nevertheless, it could be easily extended to three-dimensional location in rough scenarios.

Let us consider an acoustic event that takes place at an unknown position *x* ∈ *IR*^2^, which we wish to determine. Formally, all sensing node locations *s* = (s^1^, *s*^2^) belong to a well-defined *compact* set *X* ⊂ *IR*^2^. We denote the *Euclidean* distance *δ*(*s, x*) between a sensor location *s* and another point *x* in the scenario, *s, x* ∈ *X*, by

(1) $$\delta^{2}(s,x)={\left\|s-x\right\|}^{2}.$$

Let us also assume that a subset *B* ⊂ *X* of the nodes have detected the event, and that the position of those nodes is known. Let us denote the sound propagation speed as *υ*. Then, the time of arrival *t_b_* of the event at any node *b* ∈ *B* is:

(2) $$t_{b}=\frac{1}{\upsilon }\delta (x,s_{b})$$

and the difference between the arrival times of the event to a pair of nodes *s_b_* and *s_b_*_′_ is:

(3) $$\tau_{b-b^{\prime }}≜t_{b}-t_{b^{\prime }}=\frac{1}{\upsilon }[\delta (x,s_{b})-\delta (x,s_{b^{\prime }})],\;\forall b,b^{\prime }\in B$$

**Figure 3.**
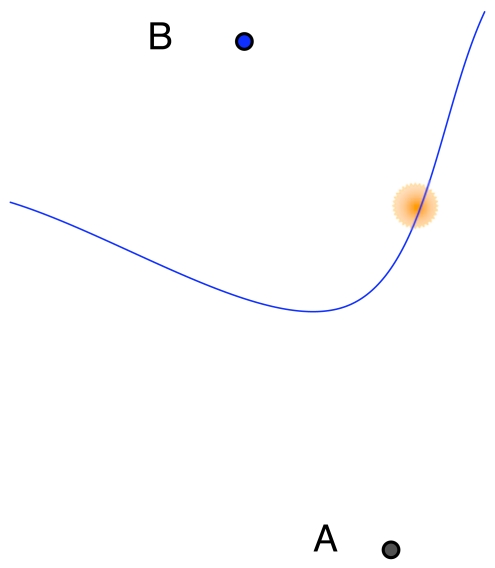

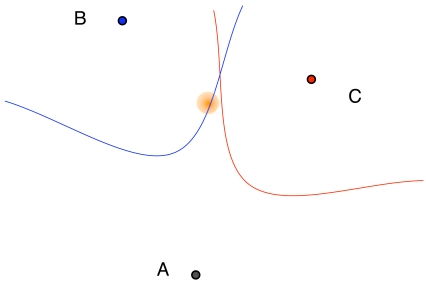
Hyperbolic multilateration of an acoustic signal. (a) Locus of the TDoA obtained between positions *x_A_* and *x_B_*. (b) Loci of the TDoA obtained between positions *x_A_* and *x_B_*, and between positions *x_A_* and *x_C_*.

This expression defines hyperboles, whose intersection determines the source of the shot. Figure 3 shows this process graphically.

For two given receiver locations, sb and *s_b′_* a set of emitter locations would yield the same TDoA measurement. This is the *locus* of possible emitter locations and it describes a hyperbole. If we now consider a receiver at a third location *s_b_*_″_, it provides a second TDoA measurement, and, hence, allows the location of the emitter on a second hyperbole. In general, a set of distances from the source to every sensor pair identifies a set of hyperboles. As a consequence, the location of the source is the intersection of this set of hyperboles. In Section 5., our tests show that location accuracy increases with the number of gunshot detection nodes. In most trials, the accuracy in scenarios with three detection nodes or more is sufficient to find the acoustic source (the detailed TDoA location technique is described in [17]).

Although non-linear expression (3) has a unique solution if there are enough hyperboles, there is some uncertainty in the calculation since:

- The speed of sound varies depending on altitude, humidity and air temperature. As we have mentioned, multi-path propagation affects the accuracy of acoustic signal detection. Single spread-spectrum techniques such as those in [27] largely mitigate it.
- The microphone directionality or polar pattern affects the result.
- The clock drift may drastically vary in time due to environmental temperature and humidity changes. In Section 2.3. we propose an approach to reduce sensor clock deviations.

Due to these inaccuracies, expression (3) may be inconsistent. Nevertheless, low-error estimations are possible. A nonlinear optimization problem can be formulated [28] to minimize the difference between estimated and real positions. We minimize the square error of the location, defined as the differences between the squares of the theoretical and measured differential arrival times to a reference node *s_b_*_′_:

(4) $$x=\arg\underset{x}{\min}\{\sum_{\forall b\in B\backslash s_{b^{\prime }}}{\left[(\delta (x,s_{b})-\delta (x,s_{b^{\prime }}))-\upsilon \tau_{b-b^{\prime }}\right]}^{2}\}$$

The least square minimization problem in (4) is not convex. Thus, standard optimization algorithms, such as incremental gradient, are not guaranteed to converge to the global minimum. The initial conditions in an iterative algorithm may lead to a local optimum or a saddle point at the termination, adding imprecision to gunshot location. The required computing power is moderate, and the GIS server (Figure 2) can handle the calculations. The solutions can be obtained practically in real time (see Section 5.). Our synchronization algorithm (see Section 2.3.) also contributes to the location procedure, since it is required for the sink node to compute the TDoA between the detectors. The tests with our reference implementation in MICAz motes reveal that, the more sensors that detect the sound event, the greater the location precision (see Section 5.). This is due to error compensation in arrival time measures. In conclusion, a high density of sensors around a sound event always improves location precision. Therefore, optimal sensor positioning improves the performance of the location system.

### 2.3. Synchronization schema

Time synchronization is a fundamental aspect in distributed sensor networks. In the proposed shot detection system, the differences in arrival times can only be computed if the nodes are tightly synchronized. To adjust the clocks, the nodes must exchange messages indicating the time reference. In fact, time adjustment degrades progressively due to clock drifts, and it is indeed mandatory to readjust it periodically (by sending new messages). However, power consumption is higher if synchronization packets are continuously transmitted. Even if a continuous energy source is available, it may be necessary to extend the network with autonomous nodes at its edges. Therefore, the number of exchanged messages should be as low possible, for a given accuracy goal. For gunshot location purposes, if we assume a maximum error of few meters, the maximum allowed error in time synchronization is in the order of a tenth of a second (*d_e_/υ*, where *d_e_* is the allowed spatial error). For example, three milliseconds of clock drift will cause an estimated error of one meter, relative to the real source position. Thus, fine-grained clock synchronization is mandatory. The tests with our synchronization algorithm show a time accuracy of tenths of microseconds. The implementation has been carried out with TinyOS in MICAz devices.

**Figure 4.**
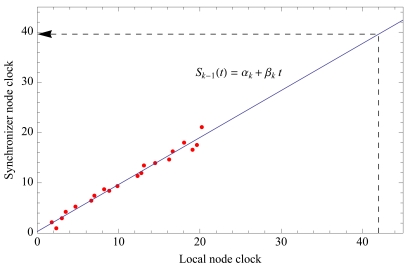
Regression line performed in a level *k* node, with several broadcast time stamps from a level *k* – 1 node.

The synchronization algorithm has two steps:

1. *Level discovery*: This step is similar to the level discovery stage in TPSN [19]. Before the synchronization process takes place, the network has to organize itself as a hierarchical tree, beginning at a root node (in our case we choose the sink). According to the minimum number of hops to the sink, a level is assigned to each node (level 0 to the root). To compute the tree, the process starts at the root, broadcasting a level discovery packet to the nodes at level 0. The nodes that receive this packet are marked as children of the root node, and they set their level to 1. The nodes ignore further level discovery packets with greater or equal level numbers. Then, level 1 nodes broadcast their level discovery packets, and so on. Note that this process also permits discover of optimal communication paths (in number of hops) to the root, and, thus, it is valid for network routing.
2. *Synchronization*: Once the hierarchical network structure is completed, the synchronization process may start. In general, level *k* nodes synchronize their children (of level *k* + 1).
  Besides its own local clock, a sensor node will maintain an estimation of its synchronizer node clock in the upper hierarchical level. The approximation consists of calculating the regression line of those two clocks. Previously, the level *k* node receives several synchronized time-stamps of level *k* – 1 (see Figure 4), which are broadcast following the tree structure that was created at the level discovery step. Figure 4 shows the regression line used to calculate the *parent* node clock in a level *k* node. Value *α_k_* represents the clock offset at reference time *t* = 0, and the slope *β_k_* is the rate of change (clock drift) of the local clock.
  Once a node detects a gunshot, it sends the event to its *parent* node in the upper level, according to the parent time clock. After one or more hops, level 0 (sink node) will receive estimations of the detection time that are synchronized with the sink clock, from one or more level 1 nodes. This way, local clock exchanges do not spend power. Since clock drift varies slowly, the regression line must only be calculated every 6 or 8 hours, according to our tests with MicaZ motes. Only large temperature variations affect the regression line slope, requiring node re-synchronization.

## 3. Optimization Model

The sound spectrum of a gunshot is dominated by the 130 Hz to 3 kHz frequency range [29]. According to [30], at these frequencies there is an extra attenuation of 3 dB each ∼25 m in woods, yielding 12 dB vs. 9 dB in open space. Given the sensitivity threshold of the sensing node, its maximum ranges are 1 Km in open space and 750 m in a wood.

We consider a large outdoor flat scenario with open space areas and wood patches. The scenario is crossed by a few power lines. In it, we wish to deploy a given number of fixed sensing nodes, so that the detection coverage is maximum. A point gets covered if it is reachable by one sensing node at least, although this capability increases if more sensing nodes see the point (to achieve source signal location we require a coverage of at least three nodes, as explained in Section 2.2.).

At the same time, we wish to minimize the distance between the sensing nodes and the power lines so that the cost of the power infrastructure is minimal in case the nodes are not autonomous. The nodes communicate through the electric grid itself, so transmission coverage is not an issue of interest.

This scenario clearly prevents an optimal educated guess, specially when only a small number of sensing nodes is available.

We define *δ*(*s, x*) = *ω*_1_(*s, x*) + *ω*_2_(*s, x*), where:

- *ω*_1_(*s, x*) corresponds to propagation distance through wood space.
- *ω*_2_(*s, x*) corresponds to propagation distance through open space.

Given a sensor location *s* ∈ *X* and a point *x* ∈ *X* we say that *x* is visible from sensor *s* if:

(5) $$\frac{1\;\text{Km}}{750\;\text{m}}\omega_{1}(s,x)+\omega_{2}(s,x)<1\;\text{Km}$$

Let *S* = {*s*_1_ …, *s_p_*}, *s_k_* ∈ *X, k* = 1, …, *p*, be the positions of *p* acoustic sensors on *X*. We denote by *V*(*x*) the set of all sensors *s* ∈ *S* that are visible from the location *x*. We also define an arbitrary grid *G* ⊆ *X.* Objective function *f*_1_, which measures acoustic sensor coverage, is defined as follows:

(6) $$f_{1}(S)=\sum_{x\in G}\{\begin{array}{ll} 0.5\cdot \text{card}(V(x)) & \text{if card}(V(x))<3\\ 3+0.01\cdot (\text{card}(V(x))-3) & \text{if card}(V(x))\geq 3 \end{array}$$

This function penalizes grid points that see fewer than three sensors (a gunshot in those points cannot be located with highest precision), and gives a small bonus to grid points that see more than three sensors (the minimum number of sensors for highest precision location).

A second objective function *f*_2_ measures the cost of the sensor deployment. As in the case of sensor coverage, there are many ways to model this. In this paper we assume that the cost of a sensor unit is negligible compared to the cost of a permanent power line. As we previously said, the scenario is crossed by *m* power lines. We define:

(7) $$f_{2}(S)=\sum_{s\in S}\underset{i\in \{1,\ldots ,m\}}{\min}\delta_{i}(s)$$

where *δ_i_*(*s*) is the Euclidean distance between *s* and the *i*-th power line, *i.e.*, between *s* and the point in that line that is closest to *s* in Euclidean distance.

Our ultimate task is to place *p* sensors on *X* in such a way that the coverage *f*_1_ on *X* is maximized and the cost *f*_2_ on *X* is minimized. Clearly, these objectives are contradictory. Minimizing −*f*_1_ (*i.e.*, maximizing *f*_1_) tends to spread the sensors, whereas minimizing *f*_2_ tends to concentrate them around the power lines. For that reason, it is desirable to produce the **Pareto front** [31] of these two functions, which represents a pool of *candidate* solutions. A point x* ∈ *X* belongs to the Pareto front of a set of functions in *X* if a further decrease in one of them is not possible without causing an increase in some of them. The methodology in [31] obtains joint descent directions for all the objective functions in a set, but it requires all of them to be differentiable, and that is not the case with *f*_1_.

In our case, since there are only two objective functions, we define the following unconstrained optimization problem:

(8) $$\underset{s_{1},\ldots ,s_{p}}{\text{minimize}}\;f(s_{1},\ldots ,s_{p})=(\theta -1)f_{1}(s_{1},\ldots ,s_{p})+\theta f_{2}(s_{1},\ldots ,s_{p})$$

By solving problem (8) repeatedly, assigning random values to *θ* in [0, 1], we obtain a collection of points of the Pareto front of *f*_1_ and *f*_2_.

### Remark 1

Note that any local minima in − *f*_1_ and *f*_2_ belong, by definition, to the Pareto front.

The next section deals with the solution of the model, including a proper choice of parameters.

## 4. Solving the Optimization Model

Many optimization algorithms are iterative. Starting with a solution estimate *S*_1_ = {s_11_, …, *s*_1_*_p_*}, a subsequence {*S_i_*}*_i_*_∈_*_I_* = {*s_i_*_1_, …, *s_ip_*}*_i_*_∈_*_I_* is generated that hopefully converges to the solution of the problem. As it is common in all implementations, there are several parameters (*magic numbers*) the user must set. Some of them will notably influence the performance of the algorithm, and often depend upon the structure of the objective function.

### 4.1. Alternative approaches

Two methods were suggested in [23, 24] for access point coverage optimization, a similar problem to ours: *neighborhood search* and *simulated annealing* [32], which we compared in [33]. These methods have no guaranteed convergence. On the other hand, gradient descent methods converge, but they can only be applied when the objective function is smooth, which is unusual in realistic models like ours.

### 4.2. Derivative-free unconstrained minimization

The function *f*(*S*), with *θ* ∈ (0, 1], is non-smooth on *X* with directional derivatives everywhere defined, which is a required assumption in a recent algorithm for unconstrained minimization (ignoring *θ* = 0 is not relevant when estimating the Pareto front, because *θ* can be arbitrarily close to 0). Numerical results show that the algorithm is competitive with others that try to find a good local minimum [25, 26]. Essentially the algorithm is an iterative process that does not force the decrease of *f*(*S_i_*), but imposes a controlled bound *φ_i_* ≥ *f*(*S_i_*) at every iteration. More specifically, given a stepsize *τ_i_*Δ > 0, and a unitary direction *D* ∈ *IR*^2^*^p^, D* = {*d*_1_, …, *d_p_*}, one iteration of the algorithm succeeds if

(9) $$f(S_{i}+\tau_{i}\Delta D)\leq f(S_{i})+\alpha_{i}(\varphi_{i}-f(S_{i}))-\nu (\tau_{i}\Delta ),$$

where *ν*(·), *φ* satisfy **A4 - A5** given below. The point *S_i_* is *blocked* when the algorithm fails to satisfy (9) on a set of directions {*D*_1_, …, *D_n_*}, *n* > 2*p* that positively spans *IR*^2^*^p^*. It is shown in [25] that under assumptions **A1 - A5** given below, the sequence of blocked points converges to a point S* that satisfies the zero order stationary point of *f*(*S*), *i.e., f′*(S*, D_k_) ≥ 0, *k* = 1, …, *n*, where the directional derivative is nonnegative along the given directions. In theory, if **A6** also holds, then ∇*f*(S*) = 0, but we are aware that **A6** is seldom fulfilled for our kind of function and we do not stress this result in this paper. The reader may read [25, 26] to complete the details. We reproduce [25, Table 1] in Table 1.

**A1.** *f*(*S*) : *IR*^2^*^p^* → *IR* is bounded below, and ${\left\{S_{i}\right\}}_{i=1}^{\infty }$ remains in a compact set,
**A2.** *f*(*S*) has directional derivatives *f′*(*S, D*) everywhere defined:
  (10) $$\eta >0\Rightarrow \{\begin{array}{l} f^{\prime }(S,\eta D)=\eta f^{\prime }(S,D),\\ f(S+\eta D)=f(S)+\eta f^{\prime }(S,D)+o(\eta ) \end{array}$$
**A3.** The unit directions *D*_1_, …, *D_n_* positively span *R*^2^*^p^*.
**A4.** The function *ν*(·) : *IR*_+_ → *IR*_+_ is *little-o* of *τ*, that is: lim *ν*(*τ*)/*τ* = 0
**A5.** The reference values ${\left\{\varphi_{i}\right\}}_{1}^{\infty }$ are upper bounds of *f*(·), *i.e., φ_i_* ≥ *f*(*S_i_*) for all *i*, and decrease sufficiently after a given finite number of successful iterations.
**A6.** *f*(*S*) is strictly differentiable or locally convex at all limit points of the sequence ${\left\{S_{i}\right\}}_{i=1}^{\infty }$ generated by the algorithm.

#### Remark 2

The simplest case of INTERPOLATE(*S, D, τ*Δ) is *Z* = *S* + *τ*Δ*D, f_Z_* = *f*(*Z*), which we follow in this paper, although there exist more elaborate alternatives [25].

#### Remark 3

If *α* = 0, we obtain the monotone version of the derivative-free algorithm, which converges to the local minimum in the neighborhood of the starting point.

#### Remark 4

**A5** holds.

#### Remark 5

In order to improve convergence, [25] suggested expanding *τ* every time a *significant* number of successes is achieved. Nevertheless, convergence is also guaranteed if *τ* only decreases.

Regarding conditions **A1-A5:** (1) The objective function *f*(·) : *IR*^2^*^p^* → *IR* as defined in (8) is non negative by adding a constant and *S* = {*s*_1_, …, *s_p_*} remains in the compact set *X*, (2) *f*(·) possesses everywhere directional derivatives if *θ* ∈ (0, 1], (3) the directions of search *D_k_* ∈ *IR*^2^*^p^, k* = 1, …, *n* are easy to generate [26], (4) the choice of *ν*(*τ*Δ) in Section 5. is 0.001(*τ*Δ)^2^, and (5) *φ* = *f*(*S*) as soon as all directions in *D* have been explored (Table 1 and Remark 4).

## 5. Numerical Tests

**Figure 5.**
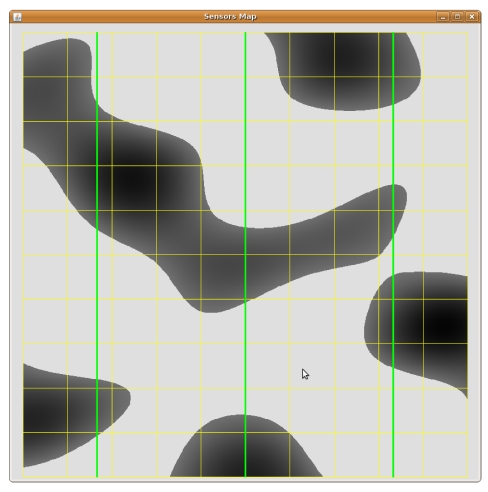
Synthetic outdoor scenario for the numerical tests. The green lines represent power lines.

We model the 10 km × 10 km flat wood scenario in Figure 5. The light areas represent open space, and the dark ones represent wood patches. The scenario is crossed by three “vertical” power lines, respectively at 1.66, 5 and 8.33 Km from the left side. This terrain representation partly encloses the difficulties in modelling Cabaneros national park.

Grid *G* is given by a uniform discretization of the scenario in 50-m steps. In it we want to install 200 acoustic sensors (*p* = 200) with maximum detection coverage, at a minimum power cost. In order to obtain the Pareto front, we obtain the solution of problem (8) several times, starting from different random points *S*_0_. We normalize the objective function by dividing it by its value at the starting points.

Since an algorithm can lead to a local optimum of problem (4) or to a solution that differs from the global optimum, we have chosen a brute force approach that computes the function at every grid point. Real-time system response is not affected, as the calculation time in a 10 km × 10 km scenario is in the order of a tenth of millisecond.

Following the results in [33], instead of applying the non-monotone derivative-free search as described in Section 4., we applied a *zone search* variant. If we simply “move” one sensor at a time, instead of “moving” them all, the evaluation of the objective function is significantly less time consuming, since most computations in *f*(*S*) do not change.

To formalize this approach, we split the scenario into *q* non-overlapping zones *X_j_, j* = 1, …, *q*, such that *X* = ∪_*j*=1..*q*_*X_j_* and *X_k_* ∩ *X_j_* = ∅, *k* ≠ *j*. Let *V*(*s_i_*) = {*x* ∈ *G* | *s_i_* ∈ *V*(*x*)}. When we move the *k*-th sensor, the remaining sensor positions do not change, *i.e.*, the group moves from *S* = {*s*_1_, …, *s_k_*, …, *s_p_*} to $S\prime =\{s_{1},\ldots ,s_{k}^{\prime },\ldots ,s_{p}\}$. When this happens, $V(S\prime )=V(S)\cup V(s_{k}^{\prime })-V(s_{k})$. To quickly obtain a *relaxed* estimate *T_v_* of $V(s_{k})\cup V(s_{k}^{\prime })$, we discard all *X_j_* | ∀*x* ∈ *X_j_* $\min(\delta (s_{k},x),\delta (s_{k}^{\prime },x))>1\;\text{Km}$. Let *T_nv_* be the set of discarded zones and let *T_v_* = *V*(*S′*) − *T_nv_*. Then, we compute Σ*_x_*_∈_*_Tv_ f*(*S′*), and keep Σ*_x_*_∈_*_Tnv_ f*(*S′*) = Σ*_x_*_∈_*_Tnv_ f*(*S*) from the previous iterate. In the numerical tests that follow, we divided our scenario in 100 [1 Km × 1 Km] zones (*q* = 100). We compute one objective function component per zone at the beginning of the algorithm execution. When only one sensor is moved, there is no need to recompute the objective function in zones that are not affected by the sensor movement.

This way, the time to compute an objective function value drops from 20 seconds on average to just 1.5 seconds on a Pentium IV.

Let *S* = {*s*_1_, …, *s_k_*, …, *s_p_*}. In order to move only one AP at a time, say *s_k_*, we generate a set of unit search directions *d_j_* ∈ *IR*^2^, *j* = 1, …, *n* such that the set *D* = {*d*_1_, …, *d_n_*} positively spans *IR*^2^. We recall that *n* ≥ 3. We declare a *success* when

$$f(s_{1},\ldots ,s_{k-1},s_{k}+\tau_{i}d_{j},s_{k+1},\ldots ,s_{p})\leq f(S)+\alpha_{i}(\varphi_{i}-f(S))-\nu (\tau_{i})$$

for some *d_j_* ∈ *D* and some *s* ∈ *S*. The point *S* is *blocked* if the algorithm is unable to move a single *s* ∈ *S*. We observe that this schema may be carried out simultaneously in a multi processor environment and it is straightforward to show that after we try all *s* ∈ *S* we have searched on a set of directions that positively span *IR*^2^*^p^*, although we are using at least 3*p* directions of search.

We tuned the method in preliminary trials and determined the following parameter values:

- Δ = 0.1 Km, *i.e.*, the method stops when the maximum sensor displacement is under 100 m.
- *ε_τ_* = 1
- *τ* = 5
- max_success= 40 (20%p), but we do not allow *τ* > 8.
- We initially set *φ* to the value of the objective function at the starting point.
- *α* = 0, *i.e.*, we perform a *monotone* derivative-free search.
- Finally, we set *ν*(*τ*Δ) = 0.001(*τ*Δ)^2^.

**Figure 6.**
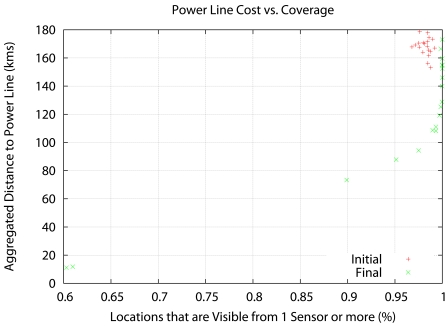
Aggregated distance to the power lines (cost) vs. coverage area with *V* (*x*) ≥ 1.

**Figure 7.**
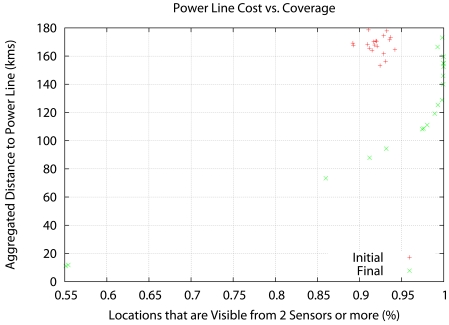
Aggregated distance to the power lines (cost) vs. coverage area with *V* (*x*) ≥ 2.

**Figure 8.**
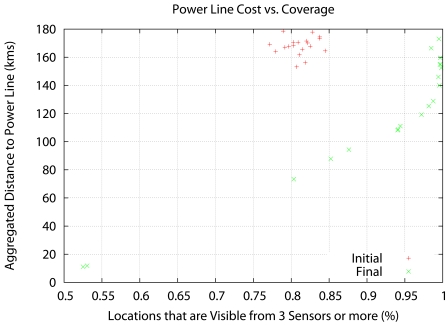
Aggregated distance to the power lines (cost) vs. coverage area with *V* (*x*) ≥ 3.

Figures 6, 7 and 8 show the results. Instead of representing *f*_2_ versus *f*_1_, we represent *f*_2_ versus the coverage areas (in percentage) that correspond to *V*(*x*) ≥ 1, *V*(*x*) ≥ 2 and *V*(*x*) ≥ 3, respectively. Algorithm execution is repeated 20 times, from 20 different starting points. Each starting point consists of a random deployment of 200 acoustic sensors in the scenario in Figure 5, with a uniform distribution.

The results reveal that the Pareto front approach is useful. The solutions show a compromise between cost and coverage. In the *V*(*x*) ≥ 1 case there is no real advantage over a random seeding in terms of coverage, although the cost drops considerably for 100% coverage. However, in the *V*(*x*) ≥ 3 case, the optimization result is clearly superior to random seeding across the whole Pareto front. At maximum cost, there is a 20% increase in coverage, and at 85% coverage (the best coverage of random seeding) there is a 50% decrease in cost.

**Figure 9.**
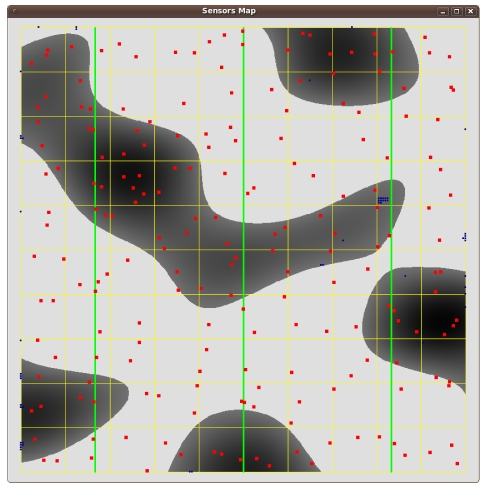
A sensor deployment for *θ* = 0.1.

**Figure 10.**
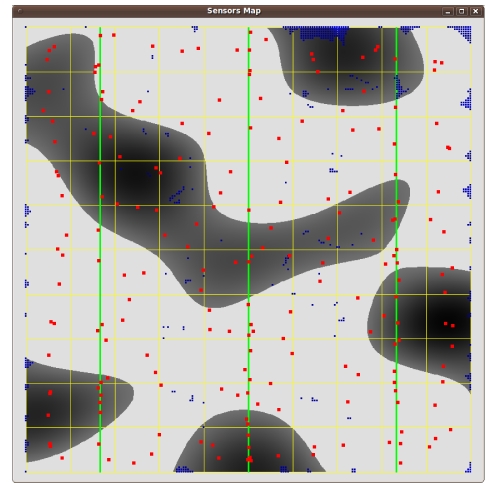
A sensor deployment for *θ* = 0.5.

**Figure 11.**
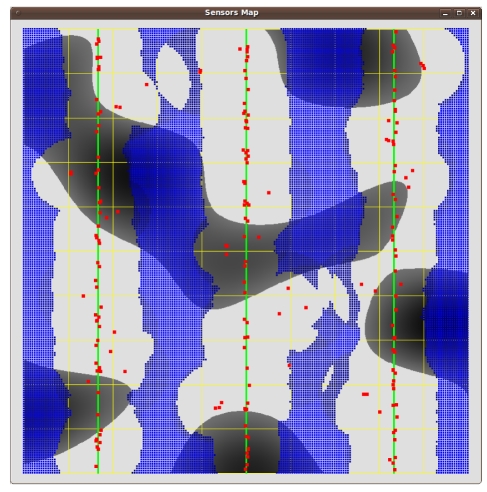
A sensor deployment for *θ* = 0.9.

Figures 9, 10 and 11 show three sensor deployments in our synthetic scenario in Figure 5, for three different choices of *θ* = {0.1, 0.5, 0.9} (red points represent sensor positions, blue areas zones with less coverage, dark blue areas indicate no coverage at all). The results are consistent with the fact that coverage improves for low *θ* values. As the values of *θ* become higher, the sensors tend to concentrate around the power lines. Therefore, power cost decreases, but so does the coverage (Figure 11 for *θ* = 0.9).

**Figure 12.**
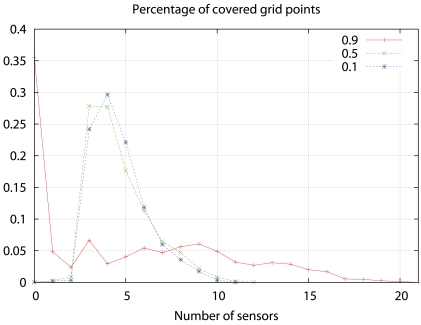
Percentage of covered grid points versus number of detecting sensors for different values of *θ*.

Figure 12 represents the percentage of grid points covered by different numbers of sensors in the three previous deployments. In the *θ* = 0.9 case, where the cost function has more weight, over 35% grid points are out of coverage, and, due to the concentration of the sensors around the power lines, there are grid points that are covered by 13 sensors or more. As *θ* decreases, grid points covered by less than 3 sensors are rare, and the majority of the grid points are covered by 3-5 sensors.

Table 2 shows location precision and expected coverage results. The location algorithm described in Section 2.2. has been tested in the three previous deployments (*θ* = {0.1, 0.5, 0.9}). Table 2 shows the average location error in meters (distance between estimated and real positions) for 10, 000 random gunshot positions in the simulation area, for each *θ* value and three levels of variation in sensor clocks. These variations are generated by adding a normal Gaussian random toss to the arrival time at each node, for three different values of *σ* between 0 and 10 ms. The results show that location accuracy increases with the number of detecting nodes, as well as with a better clock synchronization. In addition, this table provides information on the expected coverage at each scenario. For instance, in the *θ* = 0.1 case, the probability that one or more nodes detect the event is 0.99973, for two or more nodes it is 0.99777, and so on. Clearly, there must be a compromise between detection accuracy, coverage and deployment cost.

## 6. Conclusions

In this paper, we have proposed a gunshot location procedure based on sensor networks and a Pareto front approach to optimize large-scale deployments. The location procedure is based on hyperbolic multi-lateration using data from a synchronized sensor network. We also propose a practical distributed synchronization algorithm for that purpose, with low energy consumption. Sensor network planning follows a Pareto front approach, using a monotone descent method without derivatives that is compatible with realistic optimization functions. Our results are clearly superior to random placement, achieving a 50% cost reduction for 85% coverage. In the two-dimensional scenario, event detection by at least four nodes is required to achieve satisfactory gunshot location accuracy.

## Figures and Tables

**Table 1.** Non-monotone derivative-free algorithm.

**Parameters:***ε_τ_, μ, τ, φ*	
Get *S*, let *f_S_* = *f*(*S*)	
DO success= 0	
Choose *D_k_, k* = 1, …, *n* that positively span *IR*^2^*^p^*	
FOR *j* = 1 TO *n*	
[*Z f_Z_*] = INTERPOLATE (*S, D_k_, τ*Δ)	Remark 2
*α* = min(*τ, α*), *φ* = *f_S_* + *α*(*φ* − *f_S_*)	Remark 3
IF (*f_Z_* ≤ *ϕ* − *ν*(*τ*Δ))	
success=success+1	
*S* = *Z, f_S_* = *f_Z_*	
ENDIF	
END FOR	
*φ* = *f_S_*	Remark 4
IF (success>max_success)	Remark 5
*τ* = *τ* + 1	
ELSE	
*τ* = *τ* − 1	
ENDIF	
WHILE (*τ* > *ε_τ_*)	

**Table 2.** Gunshot average location error (m).

		>1 [0.99973%]	>2 [0.99777%]	>3 [0.99448%]	>4 [0.75258%]	>5 [0.45608%]

*θ* = 0.1	sinc. < 1*ms*	64.40	64.40	64.40	5.30	1.60
	sinc. 1ms	78.00	66.90	73.90	5.50	0.00
	sinc. 10ms	76.90	90.70	73.40	13.60	3.90

		>1 [0.99988%]	>2 [0.99730%]	>3 [0.98856%]	>4 [0.70991%]	>5 [0.43301%]

*θ* = 0.5	sinc. < 1*ms*	139.20	138.90	123.50	8.30	0.00
	sinc. 1ms	146.70	120.20	125.40	22.50	0.00
	sinc. 10ms	144.90	162.60	150.20	42.70	4.00

		>1 [0.64476%]	>2 [0.59659%]	>3 [0.57273%]	>4 [0.50632%]	>5 [0.47687%]

*θ* = 0.9	sinc. < 1*ms*	114.00	66.80	20.20	11.00	1.50
	sinc. 1ms	99.30	56.40	49.90	19.20	2.40
	sinc. 10ms	171.80	127.10	88.00	31.70	19.50
